# Childhood Obesity Task Forces Established by State Legislatures, 2001-2010

**DOI:** 10.5888/pcd10.120153

**Published:** 2013-08-29

**Authors:** Ashleigh L. May, Sonia A. Kim, Bettylou Sherry, Heidi M. Blanck

**Affiliations:** Author Affiliations: Sonia A. Kim, Bettylou Sherry, Heidi M. Blanck, Division of Nutrition, Physical Activity, and Obesity, National Center for Chronic Disease Prevention and Health Promotion, Centers for Disease Control and Prevention.

## Abstract

**Introduction:**

States and communities are considering policy and environmental strategies, including enacting legislation, to reduce and prevent childhood obesity. One legislative approach has been to create task forces to understand key issues and develop a course of action. The goal of this study was to describe state-level, childhood obesity task forces in the United States created by legislation from 2001 through 2010.

**Methods:**

We used the Center for Disease Control and Prevention’s Division of Nutrition, Physical Activity, and Obesity database to identify state-level childhood obesity task forces created through legislation from 2001 through 2010.

**Results:**

We identified 21 states that had enacted legislation creating childhood obesity task forces of which 6 had created more than one task force. Most task forces were charged with both gathering and reviewing information and making recommendations for obesity-prevention actions in the state. Most legislation required that task forces include representation from the state legislature, state agencies, community organizations, and community members.

**Conclusion:**

Evaluation of the effectiveness of obesity-prevention task forces and the primary components that contribute to their success may help to determine the advantages of the use of such strategies in obesity prevention.

## Introduction

The prevalence of childhood obesity in the United States is a major public health concern. Approximately 17% of children aged 2 to19 years are obese (1). Because obesity can be attributed to excess energy intake relative to insufficient energy expenditure, the majority of research and recommendations for obesity prevention has, until recently, focused on modifying an individual’s diet and physical activity. However, focusing solely on personal behaviors has not been fully successful as evidenced by increases in obesity prevalence among both adults and children in the 1990s and 2000s (2). Many environmental factors influence personal decisions and lifestyles that promote obesity. For example, many Americans, including children, live in obesogenic environments that are not conducive to making healthful food and physical activity choices (3–9). Furthermore, in certain communities and neighborhoods, inexpensive, energy-dense foods, sugary drinks, and large portion sizes are easily accessible, and affordable fruits and vegetables are not. Opportunities to engage in adequate physical activity may be limited by environmental hazards, poor neighborhood walkability, and crime. Recently, distal risk factors, such as these environmental influences have been included in the focus of obesity prevention efforts and recommendations by several organizations (10,11).

Emerging evidence suggests that policy initiatives focusing on reducing and preventing childhood obesity can support individuals’ choices and at the same time reach larger segments of the population than approaches focusing on individuals alone. This might be accomplished by promoting opportunities for active living and healthful eating (eg, making healthy choices the “optimal default” or easy choice) in communities (12,13). Legislators and the general public have begun to recognize obesity as a major public health problem (14,15). As a result, a number of policies that address aspects of childhood obesity prevention, including laws, regulations, and formal and informal rules, have been introduced in the United States at the local, state, and federal levels (16).

States and local jurisdictions have considerable influence on health-related policies through various actions, including passing laws and regulations that promote public health (17,18). Major reports such as those from the Institute of Medicine (15,19) and the US Surgeon General (20), encourage state and local governments to take active roles in considering policy as part of the solution to addressing childhood obesity (21). A first step in a number of states has been to establish task forces, councils, commissions, committees, or studies (all henceforth referred to as task forces) to examine the obesity problem in their states or to develop a course of action for additional policies and programs.

To date, the characteristics of state-level childhood obesity task forces have not been systematically examined. However, identifying and understanding the salient characteristics of legislated task forces can help determine their role and their potential impact in providing recommendations for preventing childhood obesity. Given the prevalence of childhood obesity and the considerable state government efforts related to enacting legislation on the issue, the objective of this study was to enumerate and describe the characteristics of childhood obesity task forces that were created by state legislation in the United States from 2001 through 2010.

## Methods

### Bill identification

We first identified state childhood obesity task forces that were created by state legislation from 2001 through 2010 by using 2 Web-based policy databases (22,23). We initially identified task force bills and resolutions enacted or adopted by state legislatures to address childhood obesity from the Centers for Disease Control and Prevention’s (CDC) Division of Nutrition, Physical Activity, and Obesity’s (DNPAO) database (search was conducted under previous system, now renamed, restructured, and re-released as the Chronic Disease State Policy Tracking System) (22). To identify legislation related to children and youth, we used topic area and open field search terms. The terms included task force/council, infant, infants, child, children, childhood, youth, adolescent, school, schools, childcare, daycare, Special Supplemental Nutrition Program for Women Infants and Children (WIC), and Supplemental Nutrition Assistance Program (SNAP). The CDC identifies legislation for this database systematically by using search strings specific to key obesity prevention strategies (nutrition, physical activity, and obesity). Documentation of these terms is in the State Legislative and Regulatory Action to Prevent Obesity and Improve Nutrition and Physical Activity methodology (24) and related scope notes. The search strings are applied to a legal search engine that contains information on legislation from all 50 states and the District of Columbia. Contract analysts review legislative records and compare them to scopes notes and CDC-approved decision standards to determine if they should be included in the database.

To ensure that we identified and reviewed the maximum number of legislative documents of interest, we also examined the National Conference of State Legislatures (2003–2010) (NCSL) Annual Report on Childhood Obesity Policy Options (23) as a secondary systematic source. For this search, we identified bills and resolutions based on their classification as task forces, commissions, or studies. The NCSL identified legislation through the State Net Legislative and Regulatory Information Service (http://www.statenet.com/). Keywords used to search for bills varied annually (written communication, A. Winterfeld, JD, August and October, 2012). The full text of all relevant legislation identified was obtained from individual state legislature websites.

### Data inclusion and exclusion

We identified a total of 88 bills and resolutions for review (79 from the DNPAO database and 9 from the NCSL database) and summarized them. Ten legislative documents were included in both databases, and we counted those 10 in the DNPAO database). Because they did not establish a state-level childhood obesity task force, we excluded 58 bills and resolutions; legislative documents excluded were those that focused on local-level policies only (eg, local school wellness council [n = 10]), did not focus on obesity although they may have referenced specific nutrition or physical activity prevention efforts (eg, farm to institution, community and school gardens, safe-routes-to-schools programs, assessment of general health of children [n = 23]), established general nutrition or physical activity standards (n = 8), focused only on health screenings (n = 1), amended a previous task force bill (n = 11), or did not have any association to childhood obesity (n = 5). Thus, we analyzed 30 legislative documents in the current study.

One author (A.L.M.) reviewed each of the bills and resolutions for inclusion in the study. When concerns arose regarding the inclusion or classification of legislation, a co-author reviewed it and both authors adjudicated the legislation’s inclusion in the study.

### Variables of study

Drawing on a document highlighting the importance of coalitions and partnerships to reduce obesity (25) and the criteria used to establish the mission, functions, and membership of the White House Task Force on Childhood Obesity (26), we selected 11 characteristics to examine in the 30 legislative documents (Box).

Box. Eleven Study Characteristics

**Table:** 

Characteristics	Information Extracted
Legislation type	Bill; resolution

Bill identification	State;bill number; year enacted; year terminated

Type of body established	Task force; commission; committee; council; study

Task force charge	Gather/review information (eg, study obesity or review current programs, policies, or scientific evidence); make recommendations (including reports, policies, strategic plans)

Topic area	General obesity; nutrition; physical activity

Setting	School, early care and education (ECE), community, multiple settings, not specified

Disparities addressed	Through targeted activities for high priority populations (eg, race/ethnicity, geographic location, income, disability, unspecified high priority population) in the state; through the identification of high-priority populations; through the appointment of task force members who represent the interests and organizations of, or are members of high-priority populations in the state; none addressed

Final report or recommendations	Required, not required, optional

Body member composition	Legislators; state agencies; community organizations/individual members of the community; not specified

Funding	Yes; no (excluding member compensation, per diem, reimbursement, and travel expenses)

Existence of more than one task force in the state	More than one task force; only one task force

### Results

Twenty-one state legislatures created 30 childhood obesity task forces from 2001 through 2010 (Figure). Six states created more than one task force during the study period. About half of task forces were enacted in the form of bills (n = 16) and half as resolutions (n = 14); 19 of the 30 items of legislation (63%) were enacted between 2006 and 2010. Most of the task forces created during the study period, with the exception of those created in California, Washington, and Illinois, were concentrated in Southeastern and Northeastern states.

**Figure:**
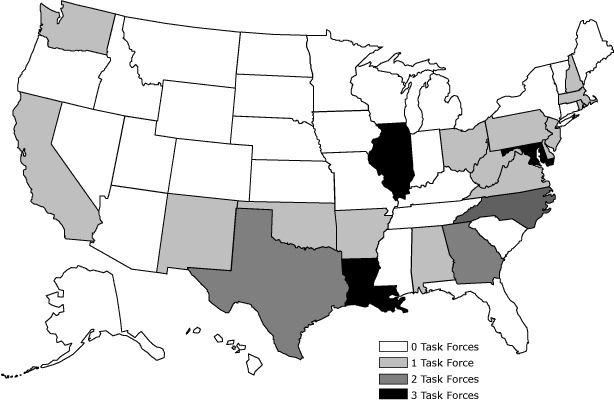
States with legislation establishing childhood obesity task forces, 2001-2010.

The primary charge (charge categories are mutually exclusive) of most task forces was to both gather and review information and to make recommendations (n = 23) followed by gathering and reviewing information only (n = 4). Most task forces were to focus on both nutrition and physical activity (n = 13) followed by general assessments of child obesity (n = 9), physical activity only (n = 5) and nutrition only (n = 3). Thirteen of the 30 task forces were to concentrate exclusively on the school setting; one examined community settings and 6 addressed multiple settings (school, community, and/or early care and education (ECE)). Ten task forces did not specify a setting. None addressed the ECE setting only (Appendix). Only 11 task forces (37%) had language specifying that their focus was to address obesity among disparate populations. Two task forces were to focus on disparities related to income (including federal nutritional programs); 6 had task force members from organizations that represented the interests of or organizations related to high priority populations or were themselves members of high priority populations. Three task forces addressed multiple disparities: one was expected to appoint members who represented high-priority populations and targeted low-income children; one was to focus on both low-income and high priority populations; and a third was to focus on economic and cultural influences associated with health and physical education.

For most task forces, members were expected to represent a cross-section of stakeholders from multiple entities, including legislative bodies, state agencies, community organizations, and individual community members. Twenty-six task forces (Table) required that the task force submit a summary report or recommendations, and 2 task forces made such reports optional. The remaining legislation made no mention of reports and recommendations. Seven legislative documents appropriated funds to complete task force activities or allowed task force members to solicit funds or develop a fund. Only 3 of the 30 task forces included information on all of the key characteristics. These task forces were established in the states of California, Texas, and Virginia (Appendix).

**Table:** Characteristics of State Childhood Obesity Task Forces Established by Legislation, United States, 2001–2010

Characteristic	n
**Legislation type^a^**	
Bill	16
Resolution	14
**Year enacted**	
2001-2005	11
2006-2010	19
**Charge^b^**	
Gather/review information only	4
Make recommendations only	3
Gather /review information and make recommendations	23
**Topic**	
Nutrition only	3
Physical activity only	5
Nutrition and physical activity	13
General assessment of obesity only	9
**Setting**	
School	13
Early care and education	--
Community	1
Multiple	6
Not specified	10
**Disparities addressed**	
Race/ethnicity	--
Geographic location	--
Income^c^	2
Disability	--
High priority population	0
Body member^d^	6
Multiple disparities	3
None	19
**Body member representation**	
Legislative body only	3
State agency only	4
Community organizations/members only	2
Legislative body and state agency	1
Legislative body and community organizations/members	1
State agency and community organizations/members	6
Legislative body plus state agency plus community organizations/ members	8
Not specified	5
**Report/recommendations required**	
Yes	26
No	2
Optional	2
**Funding^e^**	
Yes	7
No	23
**Number of task forces in state** (n = 21 states)	
States with more than one task force	6
States with only one task force	15

a A bill is defined as laws or amendments to laws. A resolution is defined as expressions of will or intent by at least one chamber of a state’s legislature.

b Charge categories are mutually exclusive.

c Includes focus on nutrition programs (eg, National School Lunch Program, Supplemental Nutrition Assistance Program (SNAP), etc.).

d Body members were from organizations that represented the interests of or organizations related to high priority populations or were themselves members of high priority populations.

e Not including member compensation, per diem, reimbursement, travel expenses.

## Discussion

To our knowledge, this exploratory study is the first to describe the characteristics and identify the content of childhood obesity task forces created through state legislation. We found that fewer than half of states enacted task forces through legislation from 2001 through 2010 to aid in their efforts to prevent or reduce childhood obesity.

Creation of more than one task force (n = 6 states) could have occurred for a number of reasons. For example, task forces may have led to additional efforts (eg, policy, environmental changes) to prevent childhood obesity or may have been found to be an effective means for addressing obesity; initial task forces may not have been effective; or states may have enacted more than one task force to address different topics, settings, or target groups. Additional research could pursue why some states created more than one task force and the subsequent impact of the task forces.

Most of the task forces created (n = 13) had language that focused solely on the school setting, perhaps because children spend about half of their waking hours in school, and because the school environment provides opportunities to learn about and practice healthful eating and physical activity, making it a prime setting for obesity prevention. Despite this, prior research has demonstrated mixed results regarding the effectiveness of school-only interventions, and suggests that comprehensive interventions that address obesity across multiple settings (eg, home, community) are more effective. For example, in a review of the literature, Shaya et al (27) found that children lost more weight in school-based interventions that included a family component than in those that focused only on the school setting. In addition to school-age children, obesity prevention is also important for young children (0-5 years) who rely on adult caretakers, often in ECE centers, to make decisions regarding opportunities for healthful eating and physical activity.

Eleven of the 30 legislated task forces addressed disparities through targeted activities for known high-priority populations in the state, through the identification of high priority populations or through the appointment of task force members who represent the interests, organizations or are members of high priority populations in the state. Obesity prevalence varies by sociodemographic factors such as income, education, sex, age, geographic location, and race/ethnicity. This leaves some population sub-groups with a higher prevalence of obesity (28). Furthermore, many of the disparities related to obesity prevalence are present early in life, which emphasizes the need for prevention efforts to begin early in childhood (29). Because most of the aforementioned risk factors (eg, age, sex, race/ethnicity) are nonmodifiable, identifying, understanding, and addressing barriers and opportunities, including culture, that influence behaviors associated with obesity among high-priority groups may help to reduce the prevalence of obesity and its consequences. Such behaviors include breastfeeding (30), physical activity (5,6), screen time (31,32), fruit and vegetable consumption (33), consumption of energy-dense foods (5,6), and sugary beverage consumption (34). Identifying and addressing the unique needs and characteristics of high-priority groups can aid state-level policymakers in making informed decisions regarding subsequent policy development and program funding. Over time, this could also aid in the development of effective, tailored interventions to reduce health disparities.

Twenty-five of the 30 task forces had language that specified who should be represented as stakeholders at the legislative, state agency, community, and organizational levels. Pomeranz (17) highlighted the importance of coordinated approaches related to obesity among state agencies. Taking this approach provides opportunities to build partnerships (35). Including diverse stakeholders in discussions related to obesity may help to better inform policymakers about the problem, to identify innovative ways to intervene across various sectors through legislation, and to identify possible co-benefits (eg, improvements in green space, reductions in food insecurity) that may increase the potential for funding for prevention activities.

Our study has limitations. First, we may not have identified all legislatively created state-level childhood obesity task forces from 2001 through 2010. Although 2 legislative databases were used to identify legislation, any relevant legislation not included in those databases would not have been captured in this study. Likewise, legislation included in the aforementioned databases may not have been captured by others. For example, a review of the Yale Rudd Center for Food Policy and Obesity database (http://www.yaleruddcenter.org/legislation/), which includes legislation beginning in 2010, found no enacted legislation related to childhood obesity task forces that met our study criteria (36). Furthermore, we did not include or examine legislation that was introduced, but not enacted, in our analyses. Finally, we included only those task forces that were legislatively created and enacted from 2001 through 2010. States may have established task forces by using other methods (eg, executive orders, nonpolicy-related actions) or established them before or after the study period we examined. Thus, this study cannot be considered an exhaustive review of all state-level childhood obesity task forces in the United States.

State-level childhood obesity task forces represent an initial step to assess the burden of obesity and to consider solutions to the problems, such as the development of policies and programs. Future research questions include “What makes for a successful state-level childhood obesity task force?” and “What is the impact of state-level childhood obesity task forces?” These questions are difficult to answer without using qualitative methodology. Locating follow-up bills and reports would be challenging at a national level because there is no available standardized data-reporting that includes outcome measures addressing the specific impact of state-level task forces or the policies that may have emerged from the task forces’ work.

In conclusion, this study provides the first known overview of components of childhood obesity task forces created by state legislation. A review of the language of the 21 state bills and resolutions establishing obesity task forces from 2001 through 2010 found significant variation in the characteristics of those task forces.

Few enacted task forces included language that focused on health disparities or health in disparate groups. However, sensitivity to cultural practices and beliefs among high-priority populations may be beneficial to help ensure that solutions are effective. A majority of task forces focused on the school setting. Healthier individual lifestyle choices could be facilitated by using systems approaches, including state-level task forces and environmental changes, in places where Americans spend their time, such as childcare facilities, schools, and worksites. Finally, most of the state task forces that were established that we studied were charged with targeting both nutrition and physical activity.

Additional research is needed to determine the effect of state-level childhood obesity task forces and the most salient characteristics associated with that effect. Furthermore, the effectiveness of task forces should be compared with other approaches to preventing obesity. Evaluation of task force effectiveness and primary components that contribute to task force success can help prioritize the focus and activities of future obesity-prevention initiatives.

## Appendix. State-Level Childhood Obesity Task Forces Established by Legislation, United States, 2001–2010

**Part I. Examples of Childhood Obesity Task Forces Established by State Legislation, 2001–2010**

Georgia

HB1580; 2004. General assessment of obesity, setting not specified; gather and review information and make recommendations.

Summary:

There is created the House Study Committee on Adult and Childhood Obesity and Prevention to be composed of 18 members. The committee shall undertake a study of the conditions, needs, and issues mentioned above or related thereto and recommend any action or legislation which the committee deems necessary or appropriate to address adult and childhood obesity and prevention in the State of Georgia.

Maryland

SB955; 2008. Physical activity; school setting; gather and review information and make recommendations.

Summary:

The Task Force shall study: 1) the advisability of requiring all public schools in the state to provide a minimum amount of physical activity or physical education to students in the public school system each week 2); the effects on childhood obesity and related health issues of requiring students to participate in a minimum amount of physical activity or physical education each week 3); the monetary costs of requiring public schools to provide a minimum amount of physical activity or physical education for students, how these costs may be minimized, and whether additional outside funding resources are available for these purposes; and 4) and analyze the results obtained by any local school systems in the State and other states that have current physical activity or physical education requirements. On or before November 20, 2008, the Task Force shall report its findings and recommendations to the Governor and, in accordance with § 2–1246 of the State Government Article, the General Assembly.

New Jersey

A3454; 2004. Nutrition and physical activity; multiple settings; gather and review information and make recommendations.

Summary:

The purpose of the task force shall be to study and evaluate, and develop recommendations relating to, specific actionable measures to support and enhance obesity prevention among the residents of this State, with particular attention to children and adolescents. The recommendations shall comprise the basis for a New Jersey Obesity Action Plan, which the task force shall present to the Governor and the Legislature pursuant to section 4 of this act.

Oklahoma

SB708;2001; Nutrition and physical activity; multiple settings; Gather and review information and make recommendations

Summary:

It shall be the duty of the task force to: 1) Study the ability of various entities, including the Governor, to raise public awareness of the problems surrounding the incidence of obesity in children (2); Investigate the feasibility of the State Board of Education creating and promoting a healthy schools initiative with awards for performance and results 3); Analyze the success of best practices models, such as ”Schools for Healthy Lifestyles” program in the Oklahoma City Public Schools (4); Ascertain quantifiable local and statewide data (5); Promote better use of various facilities, such as utilization of physical activity facilities after hours (6); Consider the feasibility of all schools participating in the Youth Risk Behaviors Survey (YRBS), and assistance to YRBS schools in developing nutrition and physical activity plans (7); Determine ways to encourage schools to offer nutritious snacks in soft drink and snack vending machines (8); Examine avenues to work with school cafeteria personnel on service of more appetizing nutritious foods; and Determine ways to encourage community-based groups or coalitions to support healthy eating and physical activity (9).

Rhode Island

SR1201;2003; General assessment of obesity; setting not specified; make recommendations

Summary:

The State of Rhode Island and Providence Plantations hereby creates a special legislative commission to make recommendations on school-age child and youth obesity prevention.

Virginia

HJ637;2007; Nutrition and physical activity; school setting; gather and review information and make recommendations

Summary:

That a joint subcommittee be established to study childhood obesity in Virginia's public schools. The joint subcommittee shall then develop legislative measures to combat the problem of childhood obesity. The joint subcommittee shall ascertain methods of combating childhood obesity in Virginia public schools and examine the relationship between the health and physical education curriculum; public health policies; social, economic, and cultural influences; media messages; and the incidence of overweight and obese students in the public schools. Further, the joint subcommittee shall examine methods to increase parental involvement and education to ensure proper nutrition of children, and survey other states to determine practices that have been useful in combating childhood obesity.

**Part II. Characteristics of 30 Childhood Obesity Task Forces Established by State Legislation, 2001–2010, by State**

Alabama

One task force legislated. Task force charged with gathering and reviewing information and making recommendations; focused on general assessment of obesity; setting not specified; addressed disparities; required reporting of activities or recommendations.

Arkansas

One task force legislated. Task force charged with making recommendations; focused on nutrition and physical activity; school setting; required reporting of activities or recommendations.

California

One task force legislated. Task force charged with gathering and reviewing information and making recommendations; focused on nutrition and physical activity; community setting; addressed disparities; required reporting of activities or recommendations; funding.

Delaware

One task force legislated. Task force charged with gathering and reviewing information and making recommendations; focused on physical activity; school setting; required reporting of activities or recommendations.

Georgia

First task force legislated. Task force charged with gathering and reviewing information and making recommendations; focused on general assessment of obesity; setting not specified; required reporting of activities or recommendations; funding.

Second task force legislated. Task force charged with gathering and reviewing information and making recommendations; focused on general assessment of obesity; setting not specified; required reporting of activities or recommendations; funding.

Illinois

First task force legislated. Task force charged with gathering and reviewing information; focused on nutrition; school setting; required reporting of activities or recommendations.

Second task force legislated. Task force charged with gathering and reviewing information and making recommendations; physical activity; school setting; required reporting of activities or recommendations.

Third task force legislated. Task force charged with gathering and reviewing information and making recommendations; physical activity; school setting; addressed disparities; required reporting of activities or recommendations.

Louisiana

First task force legislated. Task force charged with gathering and reviewing information; focused on general assessment of obesity; setting not specified.

Second task force legislated. Task force charged with gathering and reviewing information and making recommendations; focused on nutrition; setting not specified; addressed disparities; required reporting of activities or recommendations.

Third task force legislated. Task force charged with gathering and reviewing information; focused on general assessment of obesity; setting not specified; addressed disparities; required reporting of activities or recommendations.

Maryland

First task force legislated. Task force charged with gathering and reviewing information and making recommendations; focused on physical activity; school setting; required reporting of activities or recommendations.

Second task force legislated. Task force charged with gathering and reviewing information and making recommendations; focused on general assessment of obesity; setting not specified; required reporting of activities or recommendations.

Third task force legislated. Task force charged with gathering and reviewing information and making recommendations; focused on physical activity; school setting; addressed disparities; required reporting of activities or recommendations.

Massachusetts

One task force legislated. Task force charged with gathering and reviewing information and making recommendations; focused on nutrition and physical activity; multiple settings; required reporting of activities or recommendations.

New Hampshire

One task force legislated. Task force charged with gathering and reviewing information and making recommendations; focused on general assessment of obesity; setting not specified; required reporting of activities or recommendations.

New Jersey

One task force legislated. Task force charged with gathering and reviewing information and making recommendations; focused on nutrition and physical activity; multiple settings; addressed disparities; required reporting of activities or recommendations.

New Mexico

One task force legislated. Task force charged with gathering and reviewing information; focused on nutrition and physical activity; school setting.

North Carolina

First task force legislated. Task force charged with gathering and reviewing information and making recommendations; focused on nutrition and physical activity; multiple settings; required reporting of activities or recommendations; funding.

Second task force legislated. Task force charged with gathering and reviewing information and making recommendations; focused on nutrition and physical activity; multiple settings; required reporting of activities or recommendations.

Ohio

One task force legislated. Task force charged with gathering and reviewing information and making recommendations; focused on nutrition and physical activity; school setting; addressed disparities; required reporting of activities or recommendations.

Oklahoma

One task force legislated. Task force charged with gathering and reviewing information and making recommendations; focused on nutrition and physical activity; multiple settings; addressed disparities; required reporting of activities or recommendations.

Pennsylvania

One task force legislated. Task force charged with gathering and reviewing information and making recommendations; focused on nutrition and physical activity; school setting; required reporting of activities or recommendations.

Rhode Island

One task force legislated. Task force charged with making recommendations; focused on general assessment of obesity; setting not specified; required reporting of activities or recommendations.

Texas

First task force legislated. Task force charged with gathering and reviewing information and making recommendations; focused on nutrition; school setting; addressed disparities; required reporting of activities or recommendations; funding.

Second task force legislated. Task force charged with gathering and reviewing information and making recommendations; focused on general assessment of obesity; setting not specified; required reporting of activities or recommendations.

Virginia

One task force legislated. Task force charged with gathering and reviewing information and making recommendations; focused on nutrition and physical activity; school setting; addressed disparities; required reporting of activities or recommendations; funding.

Washington

One task force legislated. Task force charged with making recommendations; focused on nutrition and physical activity; school setting; required reporting of activities or recommendations.

West Virginia

One task force legislated. Task force charged with gathering and reviewing information and making recommendations; focused on nutrition and physical activity; multiple settings; required reporting of activities or recommendations; funding.
